# Characteristics of patients with acute myocardial infarction contacting primary healthcare before hospitalisation: a cross-sectional study

**DOI:** 10.1186/s12875-018-0849-8

**Published:** 2018-10-10

**Authors:** Per O. Andersson, Sofia Sederholm Lawesson, Jan-Erik Karlsson, Staffan Nilsson, Ingela Thylén, on behalf of the SymTime Study Group

**Affiliations:** 1 Primary Health Care and Department of Medical and Health Sciences, Linköping University, Linköping, Sweden; 2 Department of Cardiology and department of Medical and Health Sciences, Linköping University, Linköping, Sweden; 3 Department of Internal Medicine, Region Jönköping County, Jönköping, and Department of Medical and Health Sciences, Linköping University, Linköping, Sweden; 4 Ljungsbro Health Care Centre, Evastigen 9, 590 71 Ljungsbro, Ljungsbro, Sweden

**Keywords:** Chest pain, Myocardial infarction, Primary healthcare, Pre-hospital delay

## Abstract

**Background:**

The characteristics of patients with on-going myocardial infarction (MI) contacting the primary healthcare (PHC) centre before hospitalisation are not well known. Prompt diagnosis is crucial in patients with MI, but many patients delay seeking medical care. The aims of this study was to 1) describe background characteristics, symptoms, actions and delay times in patients contacting the PHC before hospitalisation when falling ill with an acute MI, 2) compare those patients with acute MI patients not contacting the PHC, and 3) explore factors associated with a PHC contact in acute MI patients.

**Methods:**

This was a cross-sectional multicentre study, enrolling consecutive patients with MI within 24 hours of admission to hospital from Nov 2012 until Feb 2014.

**Results:**

A total of 688 patients with MI, 519 men and 169 women, were included; the mean age was 66±11 years. One in five people contacted PHC instead of the recommended emergency medical services (EMS), and 94% of these patients experienced cardinal symptoms of an acute MI; i.e., chest pain, and/or radiating pain in the arms, and/or cold sweat. Median delay time from symptom-onset-to-decision-to-seek-care was 2:15 hours in PHC patients and 0:40 hours in non-PHC patients (*p*<0.01). The probability of utilising the PHC before hospitalisation was associated with fluctuating symptoms (OR 1.74), pain intensity (OR 0.90) symptoms during off-hours (OR 0.42), study hospital (OR 3.49 and 2.52, respectively, for two of the county hospitals) and a final STEMI diagnosis (OR 0.58).

**Conclusions:**

Ambulance services are still underutilized in acute MI patients. A substantial part of the patients contacts their primary healthcare centre before they are diagnosed with MI, although experiencing cardinal symptoms such as chest pain. There is need for better knowledge in the population about symptoms of MI and adequate pathways to qualified care. Knowledge and awareness amongst primary healthcare professionals on the occurrence of MI patients is imperative.

## Background

About 1–3% of primary healthcare (PHC) patients experience chest pain [1, 2]. Chest pain can have many different causes; most of them are of non-cardiac origin. In 10–18% of cases, the chest pain is caused by ischaemic heart disease, of which 2–4% as the result of myocardial infarction (MI) or unstable angina [3–5], conditions that require immediate attention. In some countries, general practitioners (GP) play a major role in the early care of acute MI and are often the first to be contacted by patients [6]. In most settings, however, consultation with a GP, instead of a direct call to the emergency medical services (EMS), increases pre-hospital delay [4, 7, 8]. Many cases of sudden cardiac death occur outside a hospital. Therefore, prompt action when patients experience symptoms indicating acute cardiac ischemia is of great importance [7].

According to existing European clinical guidelines on cardiovascular disease prevention, the GP should evaluate the risk factors and clinical findings when a patient contacts PHC with chest pain, and decide if the patient should be transferred to hospital [9]. However, the low prevalence of acute MI can make the diagnosis difficult [10], particularly because the medical history, symptom presentation, and findings from an electrocardiogram (ECG) are not always indicative [11, 12].

A great deal of knowledge exists today about the reasons for patient [13, 14], as well as system, delay [15]. Similarly, studies have consistently reported that only half of the patients with MI use the EMS [16, 17]. Factors associated with underutilisation of the EMS have previously been explored [18, 19]. However, the reasons for contacting the PHC for symptoms suggestive of acute MI, as a contributory factor to prolonged pre-hospital delay, have not yet been examined.

Therefore, the aims of this study were to 1) describe background characteristics, symptoms, actions and delay times in patients contacting the PHC before hospitalisation when falling ill with an acute MI, 2) compare those patients with acute MI patients not contacting the PHC, and 3) explore factors associated with a PHC contact in acute MI patients.

## Methods

### Setting

In Sweden, there are about 1300 PHC centres for a population of 10 million people. The PHC centres are staffed with approximately 6500 GPs (2000 patients per GP annually) [20]. In the Swedish emergency system, individuals with a suspected acute MI are urged to contact the EMS by telephone and describe their symptoms. Generally, when symptoms of MI are reported, an ambulance is sent. However, a substantial number of individuals experiencing MI symptoms self-transport to the ED (Emergency Department), contact their PHC or a national telephone-nurse advisement number (Swedish Healthcare Direct, SHD) as their first medical contact [8].

### Study design

This Swedish multi-centre study (SymTime) was a cross-sectional study based on self-reported data and has been described in detail previously [8]. Participants were enrolled from five different hospitals: two university hospitals and three county hospitals. The university hospitals were located in Linköping (southeast) and Umeå (northeast), and the county hospitals in Sunderby (northeast), Jönköping (south) and Kalmar (southeast), respectively. The data were collected between November 2012 and January 2014 and the hospitals were selected by geographic location and size/type of hospital.

### Participants and procedure

Within 24 hours after admittance to the coronary care unit (CCU), patients were included consecutively. The criteria for inclusion were as follows: diagnosed with acute MI [21], able to fill in the questionnaire, and willing to participate. Patients who were still clinically unstable 24 hours after admittance (i.e., with ongoing chest pain, shock or other severe symptoms) were excluded from the study, as were patients with difficulties reading and speaking Swedish. STEMI (ST-elevation myocardial infarction) patients were included at all five hospitals, while NSTEMI (non-ST-elevation myocardial infarction) patients were included at the two university hospitals.

Participants of interest were those stating in the questionnaire that they had been in contact with PHC (direct or by phone) before hospitalisation because of acute MI. In total, 694 acute MI patients were included in the SymTime study. Of those 688 (99%) had answered the question about being in contact with the PCH when falling ill. Those patients constituted the study population in the present study.

### Data collection

#### Clinical variables

The CCU nurse in charge gathered information about co-morbidities and important time point variables from the medical records, as well as from the patient. Final diagnosis (non ST-elevation MI [NSTEMI] or [STEMI]) was obtained from medical records.

#### Symptoms and pre-hospital actions

A previously validated self-administrated survey covering 35 items was used to access data on symptoms, actions, and pre-hospital delay times and transport mode to the hospital [8, 22].

### Statistical analysis

Proportions and frequencies were used to describe the patient’s characteristics and the socio-demographic, clinical and contextual variables. Continuous variables were reported as means ± standard deviation (SD) or median (25^th^ to 75^th^ percentile) as appropriate. In the bivariate analyses, we used the Pearson χ^2^ test (Fisher’s exact test when cells had expected count less than five) for categorical data and the two-tailed Student t test (for normally distributed variables) or Mann-Whitney U test (for non-normally distributed variables) for continuous data when comparing MI patients contacting the PHC (direct or by phone) before hospitalisation with all other MI patients.

A logistic multiple regression model (Enter method) was used to determine factors associated with a contact with PHC. Variables in the regression model were chosen based on results from bivariate analyses (*p*-value <. 10) or on clinical and theoretical relevance. Backward elimination was used to abort non-significant variables down to the significance level of *p*<0.05.

All tests were two tailed and a p value <0.05 indicated statistical significance. Statistical analyses were performed using SPSS software, version 25.0 (SPSS Inc, Chicago, Illinois, USA) for Windows.

## Results

### Demographics and clinical variables

A total of 688 patients with acute MI, 519 men and 169 women, were included; the mean age was 66±11 years. In total, 147 patients (21%) turned to PHC before hospitalisation when falling ill. A minority had previously experienced an MI (15%), nearly half of the participants had hypertension (47%), and 14% had diabetes. The majority (77%) were subsequently diagnosed with STEMI.

When comparing those contacting PHC before hospitalisation with patients who did not, the PHC patients more often lived in the north (i.e., more rural) areas of Sweden (46% vs. 36%, *p*<0.05) and were more often diagnosed with a NSTEMI, (31% vs. 21%, *p*<0.01). There were no significant differences with respect to age, gender, presence of time of symptom onset (time of day or weekday) or being alone or not when falling ill. The background characteristics are given in more detail in Table 1.

**Table 1:** Background characteristics of patients contacting PHC before hospitalisation compared with those not contacting PHC when experiencing symptoms of an acute MI

	All *N*=688	PHC *n*=147	Other *n*=541	*p*-value
Socio-demographics				
Age, years^b^	66 ± 11	65 ± 11	66 ± 11	0.46
Gender, men	519 (75)	112 (76)	407 (75)	0.81
Education, ≤ 9 years	270 (39)	60 (41)	210 (39)	0.68
Current smoker	174 (26)	39 (28)	135 (25)	0.60
Living alone	162 (24)	36 (25)	126 (23)	0.76
Co-morbidities^a^				
Hypertension	366 (53)	82 (56)	284 (53)	0.48
Diabetes	101 (15)	20 (14)	81 (15)	0.68
Angina Pectoris	103 (15)	21 (15)	82 (16)	0.83
Atrial fibrillation	38 (6)	6 (4)	32 (6)	0.38
Heart failure	22 (3)	1 (1)	21 (4)	**0.06**
Previous myocardial infarction	109 (16)	16 (11)	93 (17)	**0.06**
Previous stroke	26 (4)	5 (3)	21 (4)	1.00
Contextual factors				
Falling ill at home	529 (77)	110 (14)	419 (77)	0.50
Symptom onset, off hours^≠^	380 (56)	73 (50)	307 (57)	0.12
Living in the north part of Sweden	261 (38)	67 (46)	194 (36)	**0.03**
Distance to hospital, >50 km	105 (16)	22 (15)	84 (16)	0.93
Being alone at symptom onset	191 (28)	38 (26)	153 (28)	0.59
Diagnosis				
NSTEMI	160 (23)	46 (31)	114 (21)	**<0.01**

Some missing responses, which explains the differences in percentages

Data are presented as numbers (percentages) if not otherwise indicated

^a^Collected from the patients and validated against the medical records

^b^Data are presented as mean ± SD; ^≠^Evenings, nights and weekends

PHC=Primary Healthcare Centre; Other=not contacting the PHC before hospitalisation

### Symptoms when falling ill

Most of the participants experienced chest pain when falling ill (88%), and 97% experienced a combination of cardinal MI symptoms, i.e., chest pain and/or radiating pain in the arms and/or cold sweats. As well as those classic symptoms of an evolving MI, other symptoms such as weakness (39%), shortness of breath (32%), vertigo (23%) and fear (22%) were also commonly reported. The total number of symptoms reported was 5.19 (±2.49). One in five patients (*n*=141) had experienced prodromal symptoms in the previous 2 weeks. Most of the patients (66%) interpreted their acute symptoms as cardiac in origin.

When comparing those contacting PHC before hospitalisation with patients who did not, the PHC patients more seldom experienced cardinal symptoms (i.e., chest pain and/or radiating pain in the arms and/or cold sweat) (94% vs. 98%, *p*<0.05), cold sweats (44% vs. 56%, *p*<0.01), vertigo (16% vs. 25%, *p*<0.05), nausea (22% vs. 32%, *p*<0.05) or fear (15% vs. 24%, *p*<0.05). The PHC group described more worries (52% vs. 42%, *p*<0.05), a less severe pain (*p*<0.00), a lower symptom burden (*p*<0.00). There were no significant differences between groups in experiencing chest pain or prodromal symptoms. The symptoms are given in more detail in Table 2.

**Table 2:** Symptoms when falling ill in acute myocardial infarction

	All *N*=688	PHC *n*=147	Other *n*=541	*p*-value
Cardinal symptoms^a^	667 (97)	138 (94)	529 (98)	**0.03**
Symptoms, pain				
Chest pain	607 (88)	124 (84)	483 (89)	0.10
Pain in neck or throat	143 (20)	24 (16)	119 (22)	0.13
Pain in the jaw or teeth	80 (12)	18 (12)	62 (12)	0.79
Back pain	116 (16.9)	26 (18)	90 (17)	0.76
Stomach pain	58 (8)	16 (11)	42 (8)	0.23
Shoulder pain	142 (21)	34 (23)	(108 (20)	0.40
Radiating pain in the arm(s)	379 (55)	75 (51)	304 (56)	0.26
Other symptoms				
Cold sweat	368 (53)	64 (44)	304 (56)	**<0.01**
Weakness	267 (39)	54 (37)	213 (39)	0.57
Tiredness	224 (33)	45 (31)	179 (33)	0.62
Shortness of breath	219 (32)	44 (30)	175 (32)	0.58
Nausea/vomiting	206 (30)	33 (22)	173 (32)	**0.03**
Numbness in the hands	205 (30)	36 (25)	169 (31)	0.11
Vertigo	160 (23)	23 (16)	137 (25)	**0.01**
Fear	150 (22)	22 (15)	128 (24)	**0.02**
Anxiety	91 (13)	15 (10)	76 (14)	0.22
General sick feeling	103 (15)	19 (13)	84 (16)	0.43
Prodromal symptoms, ≤ 2 weeks	141 (20)	39 (27)	102 (19)	0.28
Symptom burden *	5.19 (±2.49)	4.64 (±2.19)	5.33 (±2.54)	**<0.01**
Symptom character				
Oppressive feeling across the chest	231 (38)	56 (42)	175 (38)	0.40
Dull pain	99 (16)	26 (19)	73 (16)	0.32
Tightness across the chest	85 (14)	18 (13)	67 (14)	0.77
Cramp-like pain	64 (11)	12 (9)	52 (11)	0.46
Burning pain	41 (7)	5 (4)	36 (8)	0.10
Razor-sharp pain	22 (4)	5 (4)	17 (4)	0.97
Stinging pain	21 (4)	7 (5)	14 (3)	0.28
Tenderness pain	13 (2)	2 (2)	11 (2)	0.74
Stabbing pain	6 (1)	2 (2)	4 (1)	0.62
Experience of symptoms				
Unpleasant	386 (56)	80 (54)	306 (57)	0.64
Worrying	303 (44)	77 (52)	226 (42)	**0.03**
Troublesome	191 (28)	47 (32)	144 (27)	0.22
Unbearable	188 (27)	33 (22)	155 (29)	0.14
Frightening	177 (26)	27 (18)	150 (28)	**0.02**
Anxiety-ridden	134 (20)	33 (22)	101 (19)	0.35
Tiring	138 (20)	24 (16)	114 (21)	0.25
Stressful	91 (13)	23 (16)	68 (13)	0.34
Suffocating	78 (11)	13 (9)	65 (12)	0.31
Irritating	67 (10)	15 (10)	52 (10)	0.88
Pain intensity				
Numeric rating scale^b^	6.75 (±2.04)	6.29 (±2.02)	6.87 (±2.03)	**<0.01**
Passing	22 (3)	9 (6)	13 (3)	**0.04**
Fluctuating	168 (25)	52 (36)	116 (22)	**<0.01**
Constant	359 (54)	68 (47)	291 (55)	**<0.01**
Increasing	121 (18)	16 (11)	105 (20)	**0.01**
Interpretation of the symptoms				
Cardiac in origin	456 (66)	88 (60)	368 (68)	0.08

Some missing responses, which explains the differences in percentages

Data are presented as numbers (percentages) if not otherwise indicated

^a^Chest pain and/or radiating pain in the arms and/or cold sweat; ^b^Data are presented as mean ± SD

PHC=Primary Healthcare Centre; Other=not contacting the PHC before hospitalisation

### Factors associated with contacting the primary healthcare centre

The probability of utilising the PHC before hospitalisation was associated with fluctuating symptoms (OR 1.74), pain intensity (OR 0.90), symptoms during off-hours (OR 0.42), study hospital location (OR 3.49 for Sunderby county hospital and 2.52 for Jönköping county hospital with Linköping University hospital as reference) and a final STEMI diagnosis (OR 0.58). Gender, age, co-morbidities, symptoms, interpretation of symptoms, being alone when falling ill, or distance to hospital were not significantly associated with the outcome (Table 3).

**Table 3:** Predictors of contacting the primary healthcare centre before hospitalisation^a^*n*=648

Variable	OR	95% CI	*p*-value
Diagnosis, STEMI	0.58	0.36-0.94	**0.03**
Pain intensity	0.90	0.81-0.98	**0.02**
Fluctuating symptoms	1.74	1.14-2.64	**0.01**
Symptom onset, off-hours	0.42	0.29-0.62	**<0.001**
Study hospital location (Linkoping University as reference)	1.00		**<0.01**
Jonkoping county hospital vs. Linkoping university hospital	2.52	1.32-4.82	**<0.01**
Sunderby county hospital vs. Linkoping university Hospital	3.49	1.82-6.69	**<0.01**

^a^Regression conducted using multiple logistic regression (Enter method, backward elimination). Only significant variables are presented in the final model

*OR*=Odds Ratio, *CI*=Confidence Interval, NSTEMI=non-ST elevation myocardial infarction

### Time interval from symptom onset to action

Median delay time from symptom onset to decision to seeking care was 2:15 hours in the PHC patients (P_25_ 0:30; P_75_ 11:15) and 0:40 hours (P_25_ 0:15; P_75_ 2:00) in the other patients (*p*<0.01). Twenty-three percent of the PHC patients and 6% of the other patients delayed >12 hours (*p*<0.001) before decision to seeking care. Median delay from decision until action was taken was 0:30 hours (P_25_ 0:10; P_75_ 1:07) in the PHC patients and 0:25 hours (P_25_ 0:10; P_75_ 0:40) in the other patients (*p*<0.05). When analysing the free text answers in the total group regarding reasons for not acting immediately after decision was taken, the most common reasons were; 1) waiting for the ambulance, 2) wanted to talk to a next-of-kin before going to hospital, or 3) trying self-care.

Patients that subsequently were diagnosed with STEMI (*n*=522) had a median time from symptom onset to diagnosis (i.e., ECG) of 2:02 hours (P_25_ 0:42; P_75_ 4:18). When comparing those STEMI patients contacting PHC before hospitalisation with patients who did not, those contacting the PHC had a median time from symptom onset to diagnosis of 3:15 hours (P_25_ 1:32; P_75_ 18:22) with the corresponding delay time for the non-PHC patients being 1:40 hours (P_25_1:00; P_75_ 3:19), *p*<0.001).

Reasons for not contacting the emergency medical services when falling ill

About half of the patients (53%) had previous experience of being transported by an ambulance. The reason for not contacting the EMS as a first action was predominantly because the patients did not consider themselves sick enough; this applied to 57% of the PHC patients compared with 28% of the other patients (*p*<0.00), Table 4.

**Table 4:** Reasons for not contacting the emergency medical services when falling ill

	All *N*=143	PHC *N*=42	Other *N*=101	*p*-value
*Reason for not contacting the EMS*				
Did not considered myself sick enough	52 (36)	24 (57)	28 (28)	**0.001**
My way was faster	45 (31)	12 (29)	33 (33)	0.69
Easier to take a taxi	32 (22)	6 (14)	26 (26)	0.18
Never thought about it	29 (20)	13 (31)	16 (16)	0.07
Unnecessary to call an ambulance	24 (17)	8 (19)	16 (16)	0.63
Easier to drive on my own	20 (14)	10 (24)	10 (10)	**0.03**
Others have greater needs	10 (7)	5 (12)	5 (5)	0.16
Would not like to draw attention to myself	7 (5)	0 (0)	7 (7)	0.10
Did not want to disturb the EMS	6 (4)	3 (7)	3 (3)	0.55
Thought being denied	4 (3)	1 (2)	3 (3)	1.00
Did not know the capability of the paramedics	2 (1)	1 (2)	1 (1)	0.50

*EMS*= emergency medical services, *PHC*=Primary Healthcare Centre, Other=not contacting the PHC before hospitalisation

After contacting PHC, most (80%) subsequently arrived at the hospital by ambulance; 62% contacted the EMS by themselves or with the help by a bystander.

## Discussion

The main observation of this study was that one in five people contacted PHC when experiencing symptoms suggestive of an acute MI, and this was more common in patients later diagnosed with NSTEMI. Fluctuating symptoms were predicative for PHC contact and onset of symptoms out of hours predicted other contacts than PHC. Furthermore, the PHC patients had a considerably longer delay time from symptom onset to decision to seek medical care with a median difference of 1:35 hours. A contact with the PHC also impacted on the total delay from symptom onset to diagnosis in STEMI patients. This is in line with another Swedish study that reported that patients with MI are delayed to hospital admission when they contact the PHC in the pre-hospital phase [23]. A rapid recognition and transportation of all patients with MI is crucial for their treatment. This has been well proven for STEMI, but patients with NSTEMI also benefit from reduced delay [24–26]. However, 20% of those contacting the PHC in our study did not arrive by ambulance to the hospital. For these reasons, triage of patients with chest pain is imperative. In addition to the existing clinical European guidelines on cardiovascular prevention in PHC [9], GPs are in need of validated diagnostic tools to help distinguish patients with chest pain for referral to the ED, particularly since the medical history, symptom presentation, and findings from the ECG are not always indicative [11, 27]. The use of troponin testing - a biomarker used to assess myocardial injury - may reduce emergency referrals but probably at the cost of an increased risk of overdiagnoses, especially among older patients with acute MI and often chronically increased troponin levels [2], and is therefore not encouraged in GP offices [28, 29]. However, a more in-depth medical history, detailed symptom assessment and physical examination using a clinical prediction score could possibly assist the GP to rule out coronary heart disease in PHC patients with chest pain [30, 31]. A previous study has shown that older age, male gender and a history of MI are useful predictors of ischaemic heart disease when evaluating patients with chest pain [32]. Likewise, the presence of IHD risk factors (i.e., diabetes, smoking, hyperlipidaemia) should also lead to an increased suspicion of acute cardiac ischemia, when evaluating patients with chest pain [9].

In our study, most patients contacting PHC had cardinal symptoms of MI, and no symptom was independently associated with PHC contact. Thus, symptoms do not discriminate MI patients taking contact with PHC from other MI patients. On the other hand, was symptoms waxing and waning strongly associated with PHC contact. Accordingly, a patient with cardinal MI symptoms but whose symptoms have not been constant but have instead been coming and going ought to lead to suspicion from the GP that this may be an acute MI. However, up to 80% of patients consulting a GP with chest pain have a non-cardiac diagnosis and do not need referral to the ED. Referral of all these patients would result in overcrowding of the ED [3]. Consequently, more studies are needed to discriminate factors between cardiac and non-cardiac chest pain.

We found that approximately half of the patients who contacted PHC did not consider themselves sick enough to contact the EMS. This finding is important since active care of patients with MI starts in the ambulance and the decision to claim expensive but necessary resources for transportation needs to be strengthened. Patient delay is the longest in the pre-hospital chain and a difficult aspect to modify, as previous public campaigns have shown [33], although a more recent mass media campaign in Australia demonstrated that an awareness of the campaign was significantly associated with shorter delay times [34]. Studies focusing on individual education programmes have been more successful than mass media campaigns [33, 35]. These interventions should take into account the complexity of translating knowledge into actions and tailor their message according to target groups. GPs are an important part of these pre-hospital interventions, since many patients with chest pain contact PHC. Clinicians should individualise their approach when educating patients with an increased risk of MI, such as those with hypertension, diabetes and hypercholesterolemia, and address relevant issues such as the various presentations of MI and the importance of a timely response to these symptoms [36]. Still, whatever we as clinicians do or say, some people will contact their PHC for symptoms suggestive of an acute MI, not because they are ignorant or unaware of the importance of a short delay, but just because it seems the right thing for them to do. And when they do contact the PHC, we need to respond constructively.

### Strengths and limitations

The strengths of this study are the inclusion of a large number of patients from five different hospitals in different areas of Sweden, which emphasizes the external validity of the results. There were no differences in age or gender between the groups. The aim was to include consecutive patients and there were defined criteria for inclusion in the study. A limitation of this study could be that the number of cases of NSTEMI, who are probably more often seen by a GP, was lower than the number with STEMI due to the study design. The patients were enrolled at the CCU where the number of the very oldest with multi-morbidity not suitable for interventions is usually lower than it is on general medical wards. This could mean that a healthier and younger population were included, missing the experiences of older patients with NSTEMI who may have been hospitalised in other areas or wards. One of the inclusion criteria was knowledge of the Swedish language, thus excluding, e.g., recently arrived refugees this could impact on the generalizability of the study.

## Conclusion

A substantial part of the patients contacts their PHC before they are diagnosed with MI, although experiencing cardinal symptoms such as chest pain and ambulance services are still underutilized in acute MI patients. There is need for better knowledge in the population about symptoms of MI and adequate pathways to qualified care. Knowledge and awareness amongst primary healthcare professional´s on the occurrence of MI patients is imperative.

## Abbreviations

CCU: Coronary care unit
ECG: Electrocardiogram
ED: Emergency department
EMS: Emergency medical services
GP: General practitioners
MI: Myocardial infarction
NSTEMI: Non- ST-elevation myocardial infarction
PHC: Primary healthcare
SHD: Swedish healthcare direct
STEMI: ST-Elevation myocardial infarction
